# *Aspergillus felis* sp. nov., an Emerging Agent of Invasive Aspergillosis in Humans, Cats, and Dogs

**DOI:** 10.1371/journal.pone.0064871

**Published:** 2013-06-14

**Authors:** Vanessa R. Barrs, Tineke M. van Doorn, Jos Houbraken, Sarah E. Kidd, Patricia Martin, Maria Dolores Pinheiro, Malcolm Richardson, Janos Varga, Robert A. Samson

**Affiliations:** 1 Faculty of Veterinary Science, The University of Sydney, New South Wales, Australia; 2 Department of Applied and Industrial Mycology, Centraalbureau voor Schimmelcultures- Koninklijke Nederlandse Akademie van Wetenschappen (CBS-KNAW) Fungal Biodiversity Centre, Utrecht, The Netherlands; 3 Mycology Unit, SA Pathology at Women's and Children's Hospital, Adelaide, South Australia, Australia; 4 Laboratory of Microbiology, Service of Clinical Pathology, Hospital de São João, Porto, Portugal; 5 Mycology Reference Centre Manchester, Education and Research Centre, University Hospital of South Manchester and Manchester Academic Health Science Centre, Manchester, United Kingdom; 6 Department of Microbiology, Faculty of Sciences, University of Szeged, Szeged, Hungary; Universidade de Sao Paulo, Brazil

## Abstract

We describe a novel heterothallic species in *Aspergillus* section *Fumigati*, namely *A. felis* (neosartorya-morph) isolated from three host species with invasive aspergillosis including a human patient with chronic invasive pulmonary aspergillosis, domestic cats with invasive fungal rhinosinusitis and a dog with disseminated invasive aspergillosis. Disease in all host species was often refractory to aggressive antifungal therapeutic regimens. Four other human isolates previously reported as *A. viridinutans* were identified as *A. felis* on comparative sequence analysis of the partial β-tubulin and/or calmodulin genes. *A. felis* is a heterothallic mold with a fully functioning reproductive cycle, as confirmed by mating-type analysis, induction of teleomorphs within 7 to 10 days *in vitro* and ascospore germination. Phenotypic analyses show that *A. felis* can be distinguished from the related species *A. viridinutans* by its ability to grow at 45°C and from *A. fumigatus* by its inability to grow at 50°C. Itraconazole and voriconazole cross-resistance was common *in vitro*.

## Introduction

Aspergillosis, a mycosis caused by infection with fungi belonging to the genus *Aspergillus*, occurs in a diverse range of human and animal hosts. In humans, aspergillosis is diagnosed increasingly due to the introduction of novel immunosuppressive regimens among patients undergoing bone-marrow or solid organs transplants, or treatment for malignancies [1]. Further, the prevalence of fungal infections in mammals is predicted to increase due to global warming leading to expansion of the geographic range of pathogenic fungi [2]. Invasive aspergillosis (IA) in humans occurs predominantly in the sinopulmonary tract of immunocompromised individuals after inhalation of *Aspergillus* spp. conidia. Although *Aspergillus fumigatus* is the most common cause of IA, *A. fumigatus*-like species including *A. lentulus*, *A. udagawae* (*Neosartorya udagawae*), *A. novofumigatus*, *N. pseudofischeri* and *A. viridinutans* are being increasingly identified using molecular techniques [3]–[9]. These cryptic species have reduced or variable susceptibility to antifungal drugs used for standard therapy of IA including amphotericin B and azoles, which is of concern [5], [10].

In domestic cats and dogs, in contrast to humans, fungal rhinosinusitis (FRS) is more commonly reported than invasive pulmonary aspergillosis (IPA) [11]–[12]. We recently described an emerging clinical syndrome of chronic invasive FRS, also known as sino-orbital aspergillosis (SOA), in apparently immunocompetent cats [13]. Disease is characterized by extension of a sinonasal mycosis into the orbit to form an expansive retrobulbar fungal granuloma with progressive invasion of contiguous anatomic structures including the oral cavity, subcutaneous paranasal tissues and central nervous system. Preliminary investigations suggested that the majority of infections were caused by a heterothallic *A. fumigatus*-like species. Here we identify these isolates using phenotypic, physiologic and phylogenetic analyses as *A. felis* sp. nov. and demonstrate that this species is a cause of IA in cats, dogs and humans.

## Materials and Methods

### Fungal Strains

Twenty isolates of *A. felis* were available for study including 19 from clinical specimens and 1 isolate from an indoor air sample in Germany (Table 1). Seventeen isolates were from domestic cats (15 with FRS and retrobulbar masses (SOA), 1 with sinonasal cavity infection only (sinonasal aspergillosis, SNA), 1 with a thoracic mass), one isolate was from a dog with disseminated IA and one isolate was from a human with chronic IPA. Three *A. felis* isolates, including the human isolate, had been identified and reported previously as *A. viridinutans* [14], [15]. Five additional isolates from cats with FRS or IPA were examined including two isolates of *A. fumigatus*, *A. udagawae* (1), *A. lentulus* (1) and *N. pseudofischeri* (1) (Table 1).

**Table 1:** Isolates from clinical specimens and an indoor air sample included in this study.*

Strain no./Other designation	Species	*MAT1* †	Source	GenBank accession number			
				ITS	*benA*	*CalM*	*MAT1*
DTO 131E3	*A. felis*	1	Cat, RBM, Australia	JX021671	-	-	KC797634
DTO 131E4	*A. felis*	2	Cat, RBM, Australia	JX021673	JX021692	-	KC797622
DTO 131E5	*A. felis*	1	Cat, RBM, Australia	JX021674	JX021693	JX021719	KC797627
CBS 130244	*A. felis*	1	Cat, RBM, Australia	JX021675	JX021694	JX021717	KC797630
DTO 131E9	*A. felis*	1	Cat, RBM, Australia	JX021676	JX021696	-	KC797628
DTO 131F1	*A. felis*	1	Cat, RBM, Australia	JX021677	JX021697	-	KC797629
DTO 131F2	*A. felis*	2	Cat, RBM, Australia	JX021678	JX021698	-	KC797623
DTO 131F3	*A. felis*	2	Cat, RBM, Australia	JX021679	JX021699	-	-
CBS 130245 (T) ‡	*A. felis*	2	Cat, RBM, Australia	JX021685	JX021700	JX021715	KC797620
DTO 131F6	*A. felis*	2	Cat, RBM, Australia	JX021680	JX021702	JX021721	KC797624
CBS 130246‡	*A. felis*	1	Cat, SNC, Australia	JX021681	JX021704	JX021724	KC797631
DTO 131G1	*A. felis*	2	Cat, RBM, Australia	JX021682	JX021705	JX021725	KC797625
CBS 130247‡	*A. felis*	1	Cat, RBM, Australia	JX021683	JX021706	JX021726	KC797632
CBS 130248‡	*A. felis*	2	Cat, RBM, Australia	JX021684	JX021707	JX021727	KC797621
CBS 130249	*A. felis*	2	Dog, VH, Australia	JX021686	JX021711	JX021713	-
CBS 130250	*A. felis*	1§	Cat, RBM, UK	JX021689	JX021712	JX021714	KC797633
MK 246, FRR 5679	*A. felis*	2	Cat, TM, Australia¶	-	AY590129	-	KC797626
MK 284, FRR 5680	*A. felis*	2	Cat, RBM, Australia¶	-	AY590130	-	-
MK 285, FRR 5681	*A. udagawae*	1	Cat, RT, Australia¶	-	AY590133	-	-
DTO 131F5	*A. lentulus*	1	Cat, SNC, Australia	-	-	JX021720	-
DTO 131F7	*A. fumigatus*	-	Cat, SNC, Australia	-	-	JX021722	-
DTO 131E7	*A. fumigatus*	-	Cat, SNC, Australia	-	-	-	-
DTO 131G4	*N. pseudofischeri*	1–2	Cat, SNC, Australia	-	-	JX021716	-
CM 5623	*A.felis*	1§	Human, lung, Portugal**	-	-	KC305167	-
GM 02/39††	*A.felis*	-	Human, sputum/BAL, Spain**	-	HQ127257	-	-
CM 4518††	*A.felis*	-	Human, nail, Spain	-	EU310871	-	-
IFM 54303††	*A.felis*	-	Human, Japan	-	AB248299	AB259973	-
CM 3147††	*A.felis*	-	Human, oropharyngeal exudate, Spain	-	EU310843	-	-
DTO 176F1	*A. felis*	2	Indoor air Stuttgart, Germany	-	-	KC305168	-

* DTO, internal culture collection of CBS-KNAW Fungal Biodiversity Centre, Utrecht, Netherlands; RBM, retrobulbar mass; VH, vitreous humor; TM, thoracic mass; RT, respiratory tract; SNC, sino-nasal cavity; T, type strain; BAL, bronchoalveolar lavage.

† Mating type genotype and phenotype; 1, *MAT1-1*; 2, *MAT1-2*.

‡ Isolate included in temperature growth studies.

§ Mating genotype only, negative mating test result.

¶ Previously reported as *A. viridinutans*-like [14].

** Previously reported as *A. viridinutans* in patients with IA [8], [15].

†† Sequence data only, sequences sourced from GenBank search for *A. viridinutans*.

**Figure 1:**
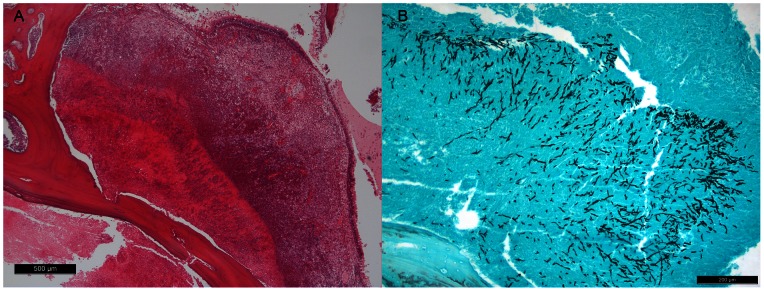
Tissue invasion by fungal hyphae in a cat with SOA. Hematoxin & Eosin- (A) and Grocott- (B) stained section of nasal mucosa and turbinates demonstrating granulomatous rhinitis (A) and submucosal invasion by septate branching fungal hyphae (B).

Except for isolates MK246, MK284 and MK285, which were obtained from the Commonwealth Scientific and Industrial Research Organisation FRR Culture Collection, North Ryde, NSW, Australia [14], all cases of invasive FRS (SOA), IA and IPA were proven invasive fungal infections based on histopathologic and/or cytopathologic detection of hyphae in needle aspiration or biopsy specimens with evidence of associated damage (Figure 1) [16]. For phylogenetic analyses, a PubMed search for *A. viridinutans* isolates from human clinical specimens deposited in GenBank with accession numbers for the internal transcribe spacer (ITS) regions, partial β-tubulin (*benA*) and/or calmodulin (*calM*) sequences was carried out. The sequences of four isolates previously identified as *A. viridinutans* (GM 02/39, CM 4518, IFM 54303, CM 3147) were identical to *A. felis* sp. nov. (Table 1). Reference strains included in the study are listed in Table S1.

### Clinical data

Cats with invasive FRS were presented by their owners for veterinary investigation of unilateral exophthalmos caused by a retrobulbar fungal granuloma. All had nasal discharge at presentation or historical evidence of sneezing or nasal discharge within the previous 6 months. The cats had no significant intercurrent diseases and were considered to be systemically immunocompetent [13]. The dog with disseminated IA presented with panuveitis, spinal pain, cardiac murmur and fever subsequent to immunosuppressive therapy with cyclosporine and prednisolone for immune-mediated hemolytic anaemia. The human patient with chronic IPA was a 56 year old man with type II diabetes mellitus receiving immunosuppressive therapy (prednisolone, etarnecept and methotrexate) for rheumatoid arthritis. Infection was protracted over 18 months and extended from a solitary lung nodule across anatomic planes to involve cervical lymph nodes and pleural space [15].

### Morphological characterization

Isolates were grown for 7 days as 3-point inoculations on Czapek agar, Czapek yeast autolysate agar (CYA), malt extract agar (MEA) and oatmeal agar (OA) at 25°C and 37°C. Media were prepared as described by Samson et al [17]. To determine *MAT1-1* and *MAT1-2* phenotypes, mating tests for teleomorph induction were performed by crossing a selection of isolates on MEA and OA in all possible combinations of opposite mating type and incubating at 30°C in the dark. Additional mating tests were performed using one isolate with confirmed phenotype and genotype of *MAT1-1* (CBS 130246) and of *MAT1-2* (CBS 130245) (Table 1). Cleistothecia produced from each positive mating were crushed and examined microscopically for the presence of ascospores. To determine ascospore viability and heat resistance 4-week old cleistothecia from two paired matings (CBS 130245× DTO 131E9, DTO 131E9× DTO131F3) were ruptured, suspended in 0.05% Tween 80 and heated to 70°C for 60 min as described previously [18]. Aliquots of ascospore suspension (200 µL) were cultured on 5% MEA, incubated at 28°C and examined by light microscopy after 18 h incubation for spore germination (Olympus BH2). Isolates were also mated with type strains of *A. viridinutans* (CBS 127.56), *A. fumigatus* (CBS 133.61) and *A. udagawae* (CBS 114217, CBS 114218).

For radial growth determination, MEA was inoculated with 3 µL of conidial suspension (3×10^7^ conidia/mL) in the centre of the plate and colony diameters were measured after 7 days incubation at temperatures ranging from 6°C in increasing increments of 3°C to 33°C, and at 36°C, 40°C, 45°C and 50°C. Morphologic features were studied by light microscopy (Olympus BH2 and Zeiss Asioskop 2 Plus) and by scanning electron microscopy (Zeiss EVO LS15). Temperature growth studies were performed using two replicates each of type strains of *A. viridinutans*, *A. fumigatus* and clinical isolates CBS 130245, CBS 130246, CBS 130247 and CBS 130248 (Table 1 & Table S1).

### Phylogeny and Molecular Identification

Isolates were grown on MEA for 7 days at 37°C and genomic DNA was extracted using the Ultraclean microbial DNA isolation kit (MoBio, Solana Beach, CA) according to the manufacturer's instructions. Amplification of the ITS regions, including ITS-1, ITS-2 and the 5.8S rDNA gene (primers ITS1 and ITS4), and parts of the β-tubulin (*benA*) (primers Bt2a and Bt2b) and calmodulin (*calM*) gene (primers cmd5 and cmd6) was performed as described previously [19]–[21]. Sequencing reactions were performed with the Big Dye Terminator Cycle Sequencing Ready Reaction kit and carried out for both strands. Sequencing reactions were purified by gel filtration through Sephadex G-50 (Amersham Pharmacia Biotech, Piscataway, NH), equilibrated in double-distilled water and analyzed on the ABI PRISM 310 Genetic Analyzer (Applied Biosystems).

Sequence alignments were performed using ClustalW software incorporated in MEGA version 5 [22]. Alignment positions with gaps or missing data were excluded, and all characters were unordered and of equal weight. The maximum parsimony (MP) tree was obtained using the Close-Neighbour-Interchange algorithm with search level 3 in which the initial trees were obtained with the random addition of sequences (10 replicates). The tree was drawn to scale with branch lengths calculated using the average pathway method. To assess the robustness of the topology, 500 bootstrap replicates were run by MP and tree length, and consistency index (CI) and retention index (RI) were calculated. *Aspergillus clavatus* (CBS 513.65) was the out-group in the trees based on *benA* and *calM* and *N. pseudofischeri* (CBS 208.92) was the out-group in the tree based on ITS sequence data. Unique ITS, *benA* and *calM* sequences were deposited in the GenBank nucleotide sequence database under accession numbers: JX021671- JX021727 and KC305167-KC305168 (Table 1).

Degenerate primers were constructed to amplify the alpha domain-encoding sequence from the *MAT1-1* gene family and the high mobility group (HMG) domain-encoding sequence from *MAT1-2*. *MAT1-1* primers (AFM1_F65655 (5′- CCT YGA CGM GAT GGG ITG G –3′) and MAT1_R6215 (5′- TG TCA AAG ART CCA AAA GGA GG –3′) were designed by identifying regions of conserved sequences in *A. clavatus*, *Neosartorya fischeri* and *A. fumigatus*. *MAT1-2* primers (MAT2_F6086 (5′- TCG ACA AGA TCA AAW CYC GTC –3) and MAT2_R6580 (5′- CTT YTT GAR CTC TTC YGC TAG G –3′) were designed by identifying regions of conserved sequence in *A. nidulans*, *N. fischeri* and *A. fumigatus*. PCR reactions were set up as described previously [23]. PCR cycle parameters were denaturation at 95°C for 5 min, then 30 cycles of denaturation at 95°C for 30 s, annealing at 48°C for 30 s, extension at 72°C for 1 min, and a final extension at 72°C for 5 min. *MAT1-1* and *MAT1-2* sequences were deposited in the GenBank nucleotide sequence database under accession numbers KC797620-KC797634 (Table 1).

### Nomenclature

The electronic version of this article in Portable Document Format (PDF) in a work with an ISSN or ISBN will represent a published work according to the International Code of Nomenclature for algae, fungi, and plants, and hence the new names contained in the electronic publication of a *PLOS ONE* article are effectively published under that Code from the electronic edition alone, so there is no longer any need to provide printed copies. In addition, new names contained in this work have been submitted to MycoBank from where they will be made available to the Global Names Index. The unique MycoBank number can be resolved and the associated information viewed through any standard web browser by appending the MycoBank number contained in this publication to the prefix http://www.mycobank.org/MycoTaxo.aspx?Link=T&Rec=. The online version of this work is archived and available from the following digital repositories: PubMed Central, LOCKSS.

### Antifungal Susceptibility Testing

Susceptibilities of 13 *A. felis* isolates to amphotericin B, itraconazole, posaconazole, voriconazole, fluconazole, 5-flucytosine, caspofungin, anidulafungin and micafungin were assessed using the Sensititre YeastOne YO10 microdilution trays (Trek Diagnostic Systems, Thermo Fisher Scientific, Australia), which have been demonstrated to yield comparable results to the CLSI M38-A standards for molds [24]. Susceptibility to terbinafine (Novartis Pharmaceuticals Corporation, Sydney, Australia) was assessed according to the CLSI M38-A2 standard [25]. Endpoints for all drugs were determined after 48 h incubation at 35°C. For amphotericin B, itraconazole, posaconazole, voriconazole, fluconazole and 5-flucytosine, endpoints were read as the lowest concentration with a complete color change, indicating total inhibition of fungal growth (minimum inhibitory concentration, MIC).The echinocandin endpoints were read as the lowest concentration revealing a partial color change, which also corresponded to the lowest concentration of drug leading to the growth of small compact hyphal forms as compared to the hyphal growth observed in the drug free control (minimum effective concentration, MEC). The terbinafine endpoint was read as the first dilution with 100% growth inhibition (MIC). *Aspergillus flavus* (ATCC 204304) and *Candida parapsilosis* (ATCC 22019) isolates were used as quality control strains.

## Results

### Sequence-based analyses

**Figure 2:**
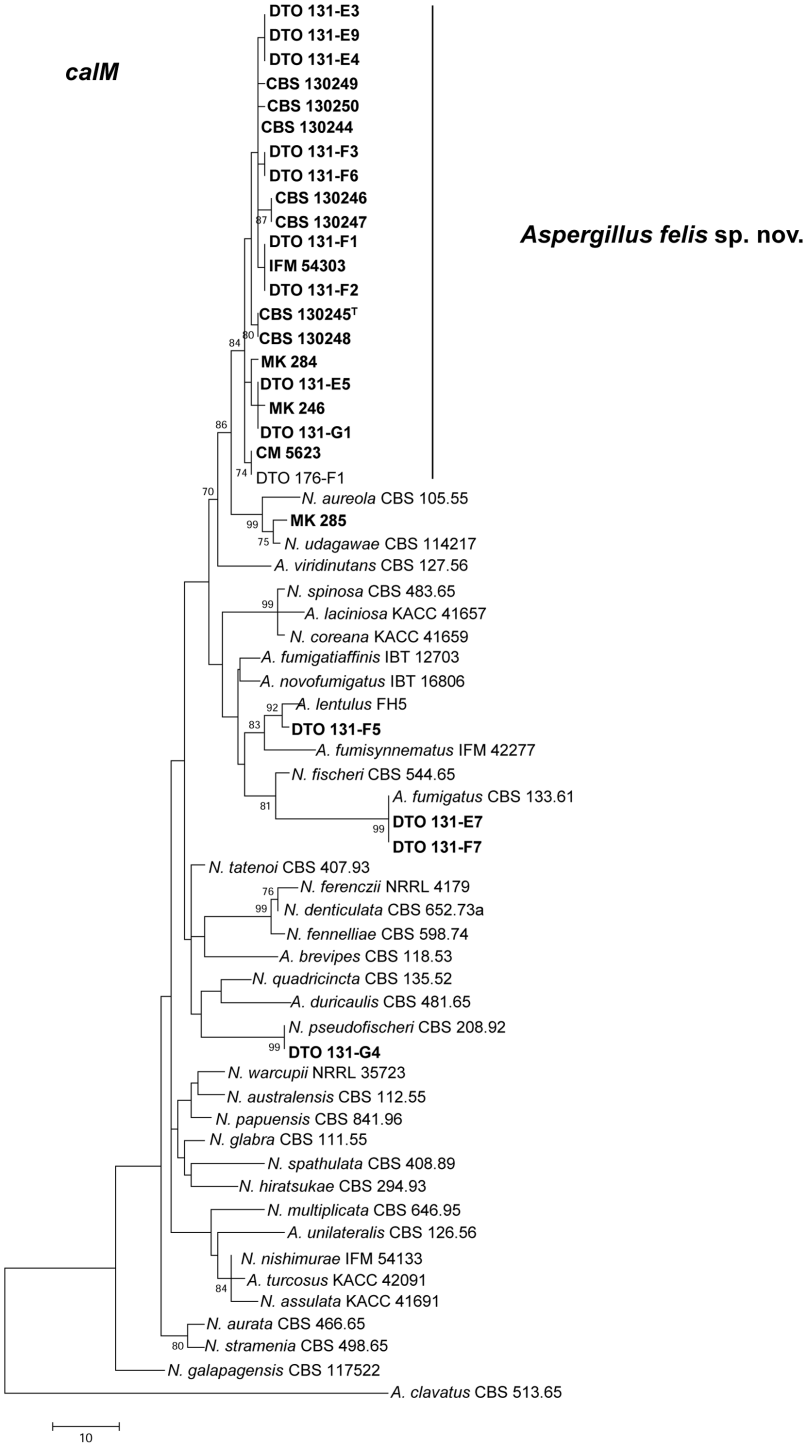
Partial calmodulin gene (*calM)* maximum parsimony (MP) tree. Phylogenetic analysis for *A. felis* sp. nov isolates and closely related species as conducted in MEGA5 [22] showing best scoring MP tree constructed using the close-neighbor-interchange algorithm [40]. Bootstrap percentages of the MP analysis are presented at the nodes for values >70%. Trees are drawn to scale, with branch lengths calculated using the average pathway method, expressed in units of the number of changes over the whole sequence. Isolates from clinical specimens used in this study are in bold. Isolate DTO 176-F1 was from an indoor air sample in Germany.

**Figure 3:**
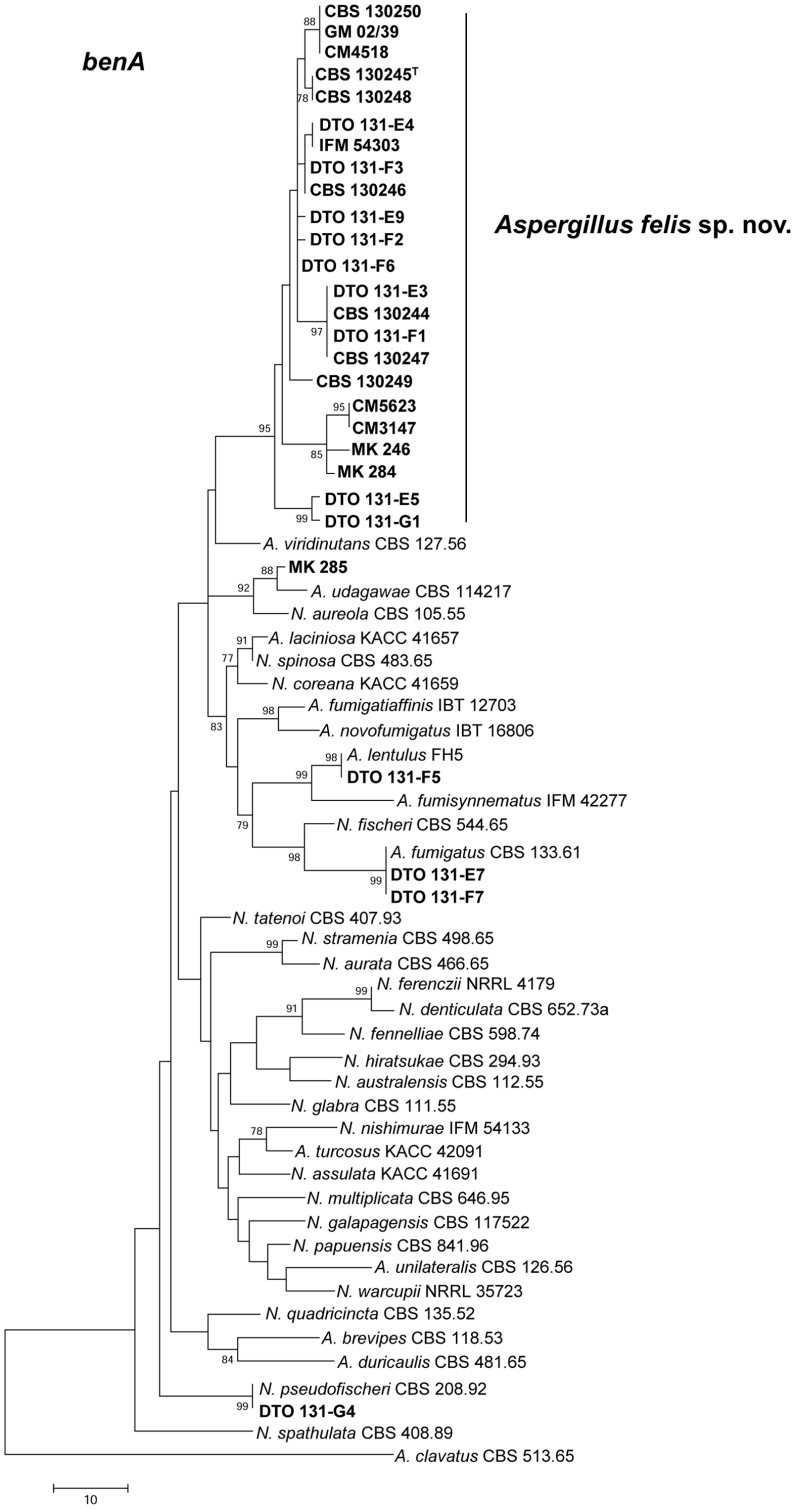
Partial β-tubulin gene (*benA*) maximum parsimony (MP) tree. Phylogenetic analysis for *A. felis* sp. nov isolates and closely related species as conducted in MEGA5 [22] showing best scoring MP tree constructed using the close-neighbor-interchange algorithm [40]. Bootstrap percentages of the MP analysis are presented at the nodes for values >70%. Trees are drawn to scale, with branch lengths calculated using the average pathway method, expressed in units of the number of changes over the whole sequence. Isolates from clinical specimens used in this study are in bold.

The *calM* data set consisted of 487 characters, including 171 parsimony informative sites. MP analysis resulted in 792 equally parsimonious trees (tree length 350 steps, CI 0.547297, RI 0.778512) (Figure 2). Of the aligned *benA* sequences, a region with 394 positions, including 63 parsimony informative characters, was selected for analysis. MP analysis resulted in 241 equally parsimonious trees (length 447, CI 0.583113, RI 0.802253) (Figure 3). The ITS data set consisted of 499 characters, including 34 parsimony informative sites. MP analysis resulted in 377 equally parsimonious trees (length 34, CI 0.800000, RI 0.947917) (Figure S1).

The tree topology inferred from *calM* grouped 17 feline isolates, the canine isolate (CBS 130249), 2 human isolates (CM 5623, IFM 54303), and the air sample (DTO 176) in a clade with 84% bootstrap support that had a most recent ancestor in common with *N. aureola* and *A. udagawae* (86% bootstrap support). *A. viridinutans* was positioned basal to these three species. Five other isolates from cats with FRS were identified as *A. fumigatus* (DTO 131-E7, DTO 131-F7), *A. udagawae* (MK 285), *A. lentulus* (DTO 131-F5) and *N. pseudofischeri* (DTO 131-G4) (Table 1, Figure 2). Analysis of *benA* showed a similar grouping as observed in the MP analysis of *calM*. In addition, three other sequences deposited in GenBank and originating from human clinical specimens (GM 02/39, CM 4518, CM 3147) belonged to the major clade comprising most clinical specimens, with high bootstrap support (95%). *A. viridinutans* was basal to this clade, but this relationship was lacking statistical support (Figure 3). On MP analysis based on ITS sequences 17 feline isolates, the canine isolate and the human isolate CM 5623 clustered together in a monophyletic group (Figure S1). All isolates belonging to this group shared identical ITS sequences. Based on the molecular data, and the phenotypic and physiological data presented below, we decided to name the strains in this clade *Aspergillus felis* sp. nov.

### Mating-type analysis

**Figure 4:**
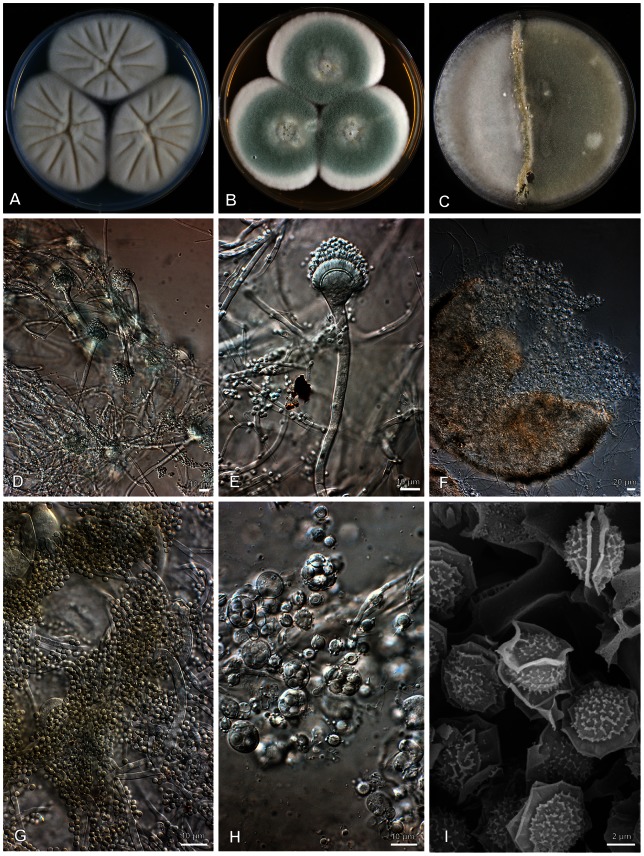
*Aspergillus felis*. Colonies growing 7 days at 25°C on CYA (A) and MEA (B); Crossing of CBS 130245 and 130246 at 30°C (C); Conidiophores and conidia **(**D, E and G); Cleistothecium (F); Ascospores (H-I).

Crosses with opposite mating partners on MEA and OA resulted in cleistothecia and ascospores within 7 to 10 days for all *A. felis* (neosartorya-morph) isolates except for CBS 130250 and CM 5623 (Table 1, Figure 4). Cleistothecia formed in small clusters mainly in the barrage zone and to a lesser extent within adjacent mycelium (Figure 4). Ascospores germinated on MEA after exposure to heat and gave rise to characteristic *A. felis* colonies. The PCR-based mating-type assay successfully amplified the fragments of the alpha- or HMG-domain genes in all *A. felis* isolates. The *MAT* genotype successfully matched the *MAT* phenotype for all isolates with positive mating tests. In addition, the PCR amplicons were sequenced (GenBank KC797620-KC797634) and a homology search on GenBank confirmed their designation to *MAT1-1* or *MAT1-*2. Mating tests with *A. udagawae, A. viridinutans* and *A. fumigatus* were negative. Immature cleistothecia containing no ascospores were observed along colony junctions for matings of *A. felis* isolates with *A. viridinutans* and *A. fumigatus*.

### Morphological and physiological characterization

**Figure 5:**
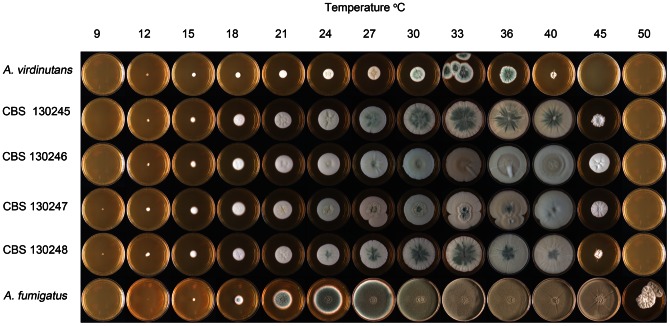
Radial growth determination at temperatures ranging from 9°C to 50°C. Type strains of *A. viridinutans* (CBS 127.56) and *A. fumigatus* (CBS 133.61) and 4 isolates of *A. felis* (CBS 130245, CBS 130246, CBS 130247, CBS 130248).

Phylogenetic analysis showed that *A. felis* is closely related to *N. aureola* and *A. udagawae*. The ascospore morphology supports this phylogenetic relationship since *A. felis*, *N. aureola* and *A. udagawae* all produce lenticular ascospores with two prominent equatorial crests and an echinulate convex surface (Figure 4) [26]. However, *A. felis* can be distinguished from *N. aureola* by its heterothallic reproduction mode and from *A. udagawae* by its ability to grow at 45°C (Figure 5), since the maximal growth temperature for *A. udagawae* is 42°C [5]. Also *A. felis* has ascospores which are 5.0–7.0×3.5–5.0 µm compared to 5–5.5×4–5 µm in *A. udagawae* [26] (Figure 4). *A. viridinutans* is phylogenetically and phenotypically related to *A. felis* and both species share the production of “nodding” conidial heads and weakly sporulating colonies (Figure 4), but can be differentiated by maximum growth temperatures. *A. felis* is able to grow at 45°C, while in our study *A. viridinutans* exhibited no growth at 45°C (Figure 5). *A. fumigatus* is phenotypically similar but is able to grow at 50°C, while none of the tested *A. felis* grew at this temperature (Figure 5).

### Species description of *A. felis*

***Aspergillus felis*** Barrs, van Doorn, Varga & Samson, **sp. nov.** [urn:lsid:indexfungorum.org:names: Mycobank MB 560382] (Figure 4).

#### Etymology

named after the first host in which clinical disease was described. *Felis* is a genus of cats in the family Felidae.

#### Diagnosis

*Aspergillus felis* (neosartorya-morph) is phenotypically similar to *A. viridinutans*, but differs by its ability to grow at 45°C. This species is phylogenetically related to *N. aureola* and *A. udagawae* and differs to *N. aureola* in having a heterothallic reproduction mode [26]–[27].

*Typus:* ex. retrobulbar mass in domestic short-haired cat, Australia (CBS H-21125-holotypus, culture ex-type: CBS 130245).

#### Description

Colonies grow rapidly on CYA agar attaining a diameter of 5.0 to 5.5 cm in 7 days at 25°C and on MEA reach 5.5 cm in diameter in 7 days at 25°C (Figure 4, panels A and B). On CYA the colony texture is mostly floccose; colonies are usually white and often sporulate poorly. On MEA colonies are more or less velvety with abundant greenish sporulation occurring after 5 to 7 days. In reverse, colonies are cream to light green. Conidiophores are uniseriate with greenish stipes and subclavate, “nodding” heads (Figure 4, panels D and E). Vesicles are subclavate with a diameter of 15–16.5 µm. Conidia are green, globose to subglobose, finely roughened and 1.5–2.5 µm in dimensions. Cleistothecia are white to creamish, 100–230 µm. Asci are globose, 8-spored, 12–16 µm in diameter (Figure 4, panel H). Ascospores are lenticular with two prominent equatorial crests and with short echinulate convex surfaces 5.0–7.0×3.5–5.0 µm (Figure 4, panel I).

#### Occurrence

This species had been found in cats with chronic invasive FRS and retrobulbar masses (SOA), IPA or with sinonasal cavity infection only (sinonasal aspergillosis, SNA), in a dog with disseminated IA, in a human with chronic IPA and in an indoor air sample in Germany.

#### Barcode

GenBank JX021685 (ITS). This species can be identified with ITS, β-tubulin and/or calmodulin sequences.

#### Taxonomy

In July 2011, the dual nomenclature system was abandoned and replaced with single name nomenclature [28]. We followed the recommendations of the International Commission on *Penicillium* and *Aspergillus* here and describe this species in *Aspergillus*, even though it produces a teleomorphic state. Using the old nomenclature rules, this species would have been included in the genus *Neosartorya*. In accordance with the Amsterdam declaration on fungal nomenclature we refer to the newly discovered teleomorph by an informal cross reference name in lower case Roman type *Aspergillus felis* (neosartorya-morph) [29].

### Antifungal susceptibilities

There was no observed activity of fluconazole or 5-flucytosine against *A. felis*. The distribution of MIC/MECs of other antifungals among those isolates that could be tested is shown in Table 2. A bimodal MIC distribution was observed for the triazoles against *A. felis*, with high MICs to at least one of the triazoles observed in four isolates including two isolates that exhibited cross-resistance to ITZ/VCZ and ITZ/VCZ/POS, respectively. Another *A. felis* isolate had a high MEC of caspofungin (2 μg/mL) but MECs of the other echinocandins were comparable to the other isolates tested within this species.

**Table 2:** Antifungal susceptibility results for 13 *A. felis* isolates from clinical specimens from cats.*

Drug	MIC/MEC (µg/mL) Distribution Among Tested Isolates										GM
	0.008	0.015	0.03	0.06	0.125	0.25	0.5	1	2	4	
AMB†						1	11	1			0.50
ITZ†			1	2	2	4	1	3			0.22
VCZ†						1	3	1	5	3	1.38
POS†			4	3	2	1	2	1			0.10
TB†						13					0.25
CSP‡	1		7	4					1		0.05
ANF‡		13									0.015
MCF‡	11	2									0.009

* MIC, minimum inhibitory concentration (†); MEC, minimum effective concentration (‡), GM, geometric mean µg/mL; AMB, amphotericin-B; ITZ, itraconazole; VCZ, voriconazole; POS, posaconazole; TB, terbinafine; CSP, caspofungin; ANF, anidulafungin; MCF, micafungin.

## Discussion

Considering the morphological, biological and phylogenetic species concepts, we present evidence for a novel species within *Aspergillus* section *Fumigati*, *A. felis* sp. nov [30]. The *BenA* and *CalM* phylogenies indicate genetic isolation of *A. felis*. We also established reproductive isolation; *A. felis* is a heterothallic fungus with a fully functioning reproductive cycle as determined by teleomorph induction and detection of *MAT1-1* and *MAT1-2* genes by PCR in isolates of corresponding phenotype. Ascospores were demonstrated to be both viable and heat resistant. The heterothallic mode of reproduction for *A. felis* should be confirmed further by the establishment of meiotic recombination in ascospore progeny as was described by O'Gorman and Dyer in their parallel discovery of a functional heterothallic sexual cycle in *A. fumigatus* [18]. No teleomorph has as yet been isolated *in vitro* for *A. viridinutans* although results of mating-type genotype determination in our study indicate it is a heterothallic fungus. Cryptic sexual states are being increasingly identified in mitosporic fungi [31]. For heterothallic fungi a strong correlation has been established between biological species based on mating tests and phylogenetic species based on multilocus sequence typing [32]. Differences in maximum growth temperature were also identified between *A. felis* and the type strain of *A. viridinutans*. *A. felis* is a thermotolerant fungus, as defined by a maximum growth temperature of ≥45°C and a minimum growth temperature of <20°C. The maximal growth temperature of both *A. viridinutans* and *A. udagawae* has been reported as 42°C [5], [33].

Species within *Aspergillus* section *Fumigati* cannot be reliably identified on the basis of morphologic criteria alone [4]. In contrast to other *Aspergillus* species, *A. felis* can be reliably identified with ITS sequences only. Molecular identification of *A. fumigatus*-like molds in human patients with IA has important clinical relevance since clinical disease characteristics vary with infecting species and MICs of antifungal drugs, especially triazoles, are often high [5]–[7], [10], [34]. This is exemplified in a study of 86 isolates from patients with IA identified as *A. fumigatus* on phenotypic features. When the same isolates were later identified by sequencing the partial *benA* and rodlet A genes, 12 (14%) were identified as *A. udagawae* [5]. In patients with *A. udagawae* infection, the median duration of illness was 7 times longer than in patients with confirmed *A. fumigatus* infection, disease was refractory to standard therapy and MICs of various antifungals of *A. udagawae* isolates were higher than those for *A. fumigatus* [35]. Similarly, a distinctive form of IA characterized by chronicity, propensity to spread across anatomical planes and reduced antifungal susceptibility was attributed to infection with *A. viridinutans* based on comparative partial *benA* and rodlet A gene sequence analyses [15]. We re-examined this isolate (CM 5623) and our molecular data show that it belongs to *A. felis*. On our phylogenetic analyses, the partial *BenA* and/or *CalM* sequences of four other human isolates from clinical specimens identified previously as *A. viridinutans* signified identity with *A. felis* [33]–[34]. One of these (GM 02/39) was from a patient with IA and co-infection with *A. novofumigatus*, another *A. fumigatus* sibling species [8]. These findings are strongly supportive of a pathogenic role for *A. felis* in IA in humans. It is likely that analysis of other clinical isolates identified worldwide as *A. viridinutans* would reveal more strains of *A. felis*; determining the geographical distribution and prevalence of this species as an emerging fungal pathogen would be of interest. Our finding that *A. felis* was present in an air sample in Germany indicates that this species can be distributed by airborne propagules. This strain readily produced cleistothecia within 7 days after mating with CBS 130246 (*MAT1-1*).

**Figure 6:**
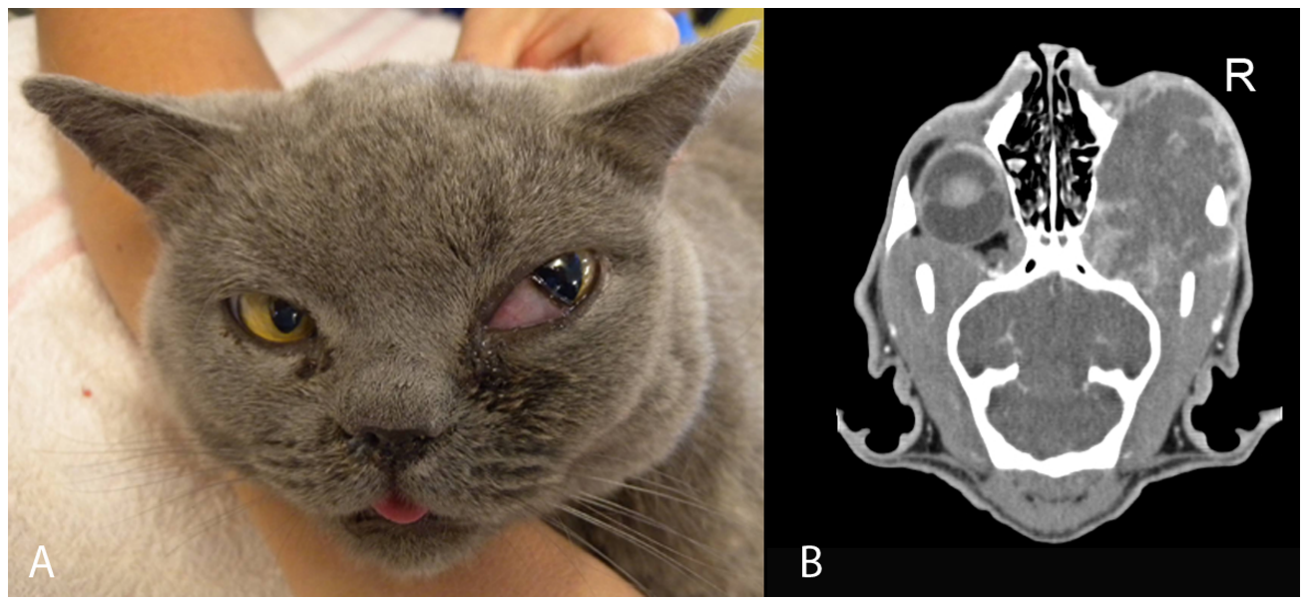
Cat with sino-orbital aspergillosis (invasive fungal rhinosinusitis) caused by A. felis with exophthalmia and prolapse of the nictitating membrane (third eyelid) associated with a retrobulbar fungal granuloma (A). Coronal CT scan soft-tissue post-contrast view showing retrobulbar fungal granuloma occupying the inferior aspect of the orbit with involvement of the adjacent paranasal subcutaneous tissues (B).

Similar to chronic atypical IPA of humans, *A. felis* infection in feline invasive FRS has a protracted clinical course and spreads across anatomical planes to involve contiguous tissues (Figure 6). Domestic cats, the most common host described with *A. felis* infection, represent a suitable model for translational research of naturally occurring IA caused by *A. fumigatus*-like molds in immunocompetent hosts. In contrast to disease in humans, mycoses such as aspergillosis and cryptococcosis typically cause rhinosinusitis rather than pulmonary infection in cats. This may be associated anatomical differences in the nasal cavity and paranasal sinuses resulting in preferential deposition of inhaled fungal spores within the sinonasal cavity in cats compared to the lower respiratory tract in humans. Most isolates in the current study were obtained from cats with sino-orbital aspergillosis that failed to respond to aggressive multimodality therapy including posaconazole, amphotericin-B and, in some cases, radical orbital debridement surgery (exenteration) [13]. The majority were euthanased due to disease progression with severe signs including blindness and generalised seizures. IPA in the two human patients from which *A. felis* was isolated, was similarly refractory to treatment with posaconazole, voriconazole and caspofungin or liposomal amphotericin-B and caspofungin respectively [8], [15]. Infections were fatal in both cases. In addition to *A. felis,* we have demonstrated that other species in the *Aspergillus* section *Fumigati* cause FRS in domestic cats including *A. fumigatus*, *A. udagawae*, *A. lentulus* and *N. pseudofischeri*.

Epidemiological cut off values (ECV) of amphotericin B for several *Aspergillus* species have recently been determined using the CLSI broth microdilution method [36]. The modal MIC of amphotericin B for 3, 988 *A. fumigatus* isolates was 0.5 µg/mL and the ECV that captured 95% of the modelled wild-type population was ≤2 µg/mL. MICs of amphotericin B were ≤1 µg/mL for all 13 *A. felis* isolates tested in our study and were ≤0.5 µg/mL for 12 of these. By contrast, three other cryptic species *A. lentulus, A. udagawae* and *A. fumigatiaffinis* have comparatively high MICs of amphotericin B [4], [34]. Elevated MICs of amphotericin B have been associated with poor clinical outcomes for infections caused by *A. terreus* and *A. udagawae* [35], [37]. Including antifungal susceptibility data published previously for human *A. felis* isolates CM 5623, CM3147, CM4158 and GM 02/39, high MICs of voriconazole (4 µg/mL) and/or itraconazole (≥1 µg/mL) occurred in 41% (7/17) of *A. felis* isolates [8], [15], [34]. Similar susceptibility trends have been observed in *N. pseudofischeri* [34]. However, where azole cross resistance was identified in *A. fumigatus* isolates it was usually between itraconazole and posaconazole (54%) and was uncommon between itraconazole and voriconazole (7%) [38]. High MICs of itraconazole (>16 µg/mL) and voriconazole (4 µg/mL) correlated with clinical outcome in one case of *A. felis* IA in a human (isolate CM5623) where sequential administration of these failed to control disease progression [15]. Additionally, one feline isolate with a caspofungin MEC of 2 µg/mL in this study, along with two human isolates with MEC of 1 µg/mL reported previously [8], [34] suggests variable susceptibility of *A. felis* to caspofungin *in vitro*. Caspofungin ECVs accounting for 95% and 99% of the modelled *A. fumigatus* population were set at 0.5 µg/mL and 1 µg/mL, respectively [39]. The antifungal susceptibility profile of *A. felis* indicates that it is another *A. fumigatus­*-like mold with *in vitro* resistance to antifungal agents used routinely for prophylactic therapy and treatment of IA.

## Conclusion

*A. felis* is an important new species in *Aspergillus* section *Fumigati* applying a polyphasic taxonomical approach including molecular, morphological, physiological and ecological data. *A. felis* is an emerging agent of invasive aspergillosis in cats, dogs and humans.

## Supporting Information

Figure S1 Phylogenetic analysis of the ITS gene for *A. felis* sp. nov isolates and other closely related species as conducted in MEGA5 [22] showing best scoring maximum parsimony (MP) trees constructed using the close-neighbor-interchange algorithm [40]. Bootstrap percentages of the MP analysis are presented at the nodes for values >70%. Trees are drawn to scale, with branch lengths calculated using the average pathway method, expressed in units of the number of changes over the whole sequence.
(TIF)

Table S1 **Genbank accession numbers for genes of additional isolates used in morphologic and/or phylogenetic analyses in this study.**
(DOC)
