# Supplementary figures and images for: Aspergillus felis sp. nov., an Emerging Agent of Invasive Aspergillosis in Humans, Cats, and Dogs
Source: PLoS One. 2013 Jun 14;8(6):e64871. doi: 10.1371/journal.pone.0064871 (PMC3683053; doi:10.1371/journal.pone.0064871)

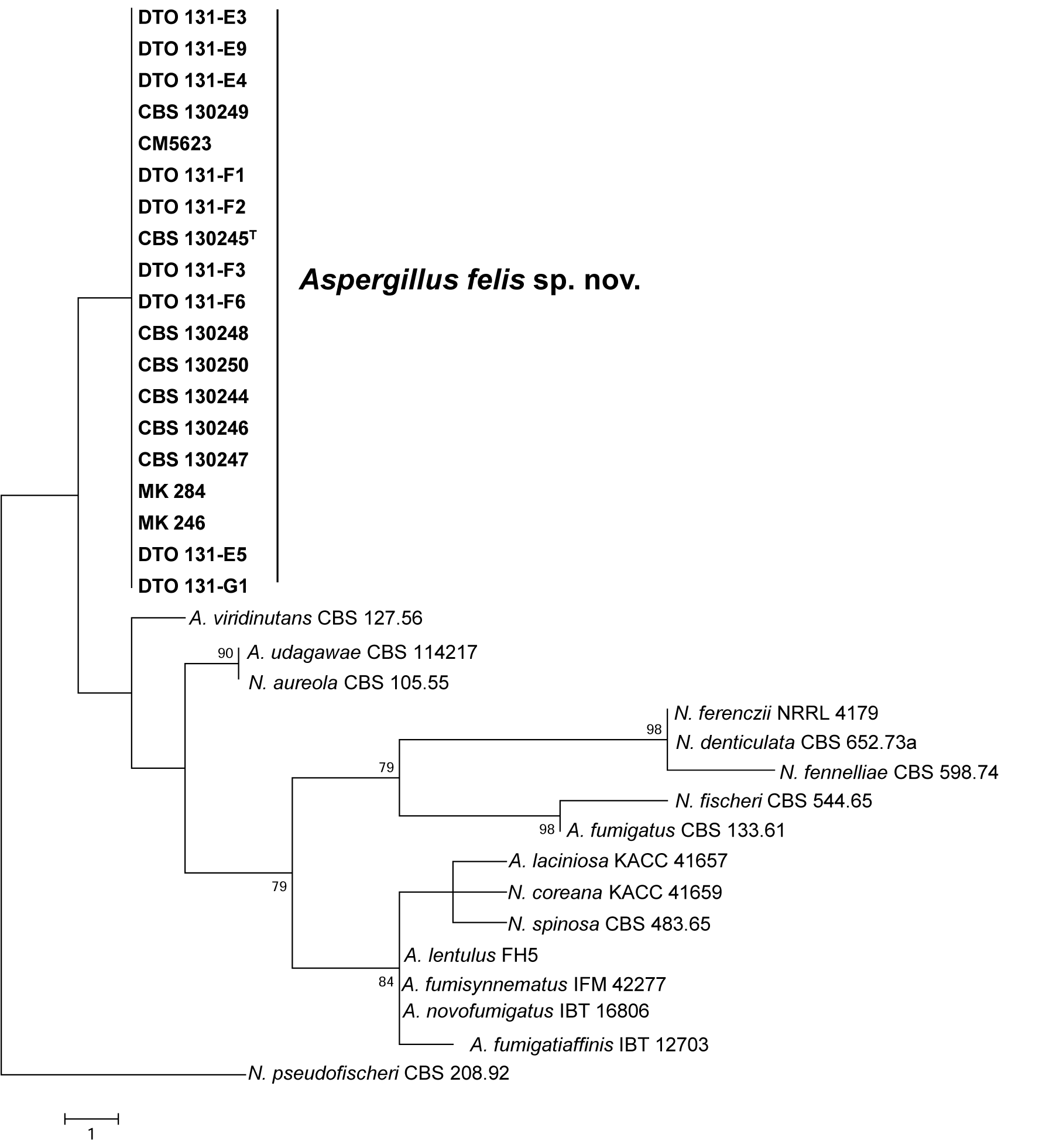

Supplement: Figure S1 — Phylogenetic analysis of the ITS gene for A. felis sp. nov isolates and other closely related species as conducted in MEGA5 [22] showing best scoring maximum parsimony (MP) trees constructed using the close-neighbor-interchange algorithm [40]. Bootstrap percentages of the MP analysis are presented at the nodes for values >70%. Trees are drawn to scale, with branch lengths calculated using the average pathway method, expressed in units of the number of changes over the whole sequence. (TIF) [file pone.0064871.s001.tif]
