# Supplementary material for: Aspergillus felis sp. nov., an Emerging Agent of Invasive Aspergillosis in Humans, Cats, and Dogs
Source: PLoS One. 2013 Jun 14;8(6):e64871. doi: 10.1371/journal.pone.0064871 (PMC3683053; doi:10.1371/journal.pone.0064871)
Supplement: Table S1 — Genbank accession numbers for genes of additional isolates used in morphologic and/or phylogenetic analyses in this study. (DOC) [file pone.0064871.s002.doc]

**Supporting Information Table S1. Genbank accession numbers for genes of additional isolates used in morphologic and/or phylogenetic analyses in this study**

| Strain | Other designation | Isolate | Source | GenBank accession no. | | | Reference |
| --- | --- | --- | --- | --- | --- | --- | --- |
|  |  |  |  | *benA* | *calM* | ITS |  |
| CBS 118.53 | NRRL 2439 | *A. brevipes,* T* | Soil, Australia | EF669812 | EF669881 | - | [1] |
| CBS 513.65 | NRRL 1656 | *A. clavatus*, T | unknown | EU076340 | EU078665 | - | [2] |
| CBS 481.65 | NRRL 4021 | *A. duricaulis*, T | Soil, Argentina | EF669827 | EF669897 | - | [1] |
| IBT 12703 | CBS 117186 | *A. fumigatiaffinis*, T | Soil, USA | DQ094885 | DQ094891 | AB299410 | [3] |
| CBS 133.61 | NRRL 163 | *A. fumigatus,* T | Chicken lung, USA | AY685150 | AY689334 | HQ026746 | [3] |
| IFM 42777 |  | *A. fumisynnematus*, T | Soil, Venezuela | AB248076 | AB259968 | AB185274 | [4] |
| KACC 4167 | CBS 11721 | *A. laciniosa*, T | Soil, Korea | AY870756 | AY870716 | - | [5] |
| FH5 | CBS 117887 | *A. lentulus,* T | Human clinical specimen, USA | AY738513 | DQ094896 | AY738515 | [6] |
| IBT 16806 | CBS 117520 | *A. novofumigatus*, T | Soil, Ecuador | DQ094886 | DQ094893 | AB299411 | [3] |
| KACC 42091 | IBT 27921 | *A. turcosus*, T | Air conditioner, Korea | DQ534143 | DQ534148 | - | [7] |
| CBS 126.56 | KACC 41140 | *A. unilateralis*, T | Rhizosphere, Australia | AF057316 | AY689366 | - | [3] |
| CBS 114217 | CBM-FA 0702 | *A. udagawae,* T | Soil, Brazil | AF132226 | AY689372 | AB250781 | [8] |
| CBS 127.56† | NRRL4365 | *A. viridinutans*, T | Rabbit dung, Australia | EF661252 | EF661266 | EF661280 | [1] |
| KACC 4161 |  | *N. assulata*, T | Soil, Korea | DQ114123 | DQ114131 | - | [7] |
| CBS 466.65 |  | *N. aurata*, T | Soil, Brunei | AF057318 | AY870685 | - | [7] |
| CBS 105.55 | NRRL 2244 | *N. aureola,* T | Soil, Ghana | EF669808 | EF669877 | EF669950 | [1] |
| CBS 112.55 |  | *N. australensis,* T | Soil, Australia | AY870739 | AY870698 | - | [5] |
| KACC 41659 | NRRL 35590 | *N. coreana,* T | Soil, Korea | AY870758 | AY870718 | AB299414 | [5] |
| CBS 652.73a | KACC 41183 | *N. denticulata*, T | Soil, Suriname | DQ114125 | DQ114133 | EU220282 | [7] |
| CBS 598.74 |  | *N. fennelliae* | Rabbit, clinical specimen | DQ114127 | DQ114135 | EF669994 | [7] |
| NRRL 4179 | CBS 121594 | *N. ferenczii* | Soil, Australia | EF669833 | EU220285 | EU220282 | [9] |
| CBS 544.65 | NRRL 181 | *N. fischeri,* T | Canned apples | AF057322 | AY689370 | AB185254 | [1] |
| CBS 117522 | IBT 16757 | *N. galapagensis,* T | Soil, Ecuador | DQ534145 | DQ534151 | - | [7] |
| CBS 111.55 |  | *N. glabra,* T | Rubber tire, USA | AY870734 | AY870693 | - | [7] |
| CBS 294.93 | NRRL 20819 | *N. hiratsukae,* T | Aloe juice, Japan | AF057324 | AY870699 | - | [10,5] |
| CBS 646.95 |  | *N. multiplicata*, T | Soil, Taiwan | DQ114129 | DQ114137 | - | [7] |
| IFM 54133 |  | *N. nishimurae,* T | Soil, Kenya | AB201360 | HE974392 | - | - |
| CBS 841.96 |  | *N. papuensis*, T | *Podocarpus* tree bark, PNG | AY870738 | AY870697 | - | [9] |
| CBS 208.92 | NRRL 20748 | *N. pseudofischeri*, T | Human clinical specimen, USA | AY870742 | AY870702 | EF669946 | [5] |
| CBS 135.52 | NRRL 2154 | *N. quadricincta,* T | Cardboard, UK | EF669806 | EF669875 | - | [1] |
| CBS 408.89 | NRRL 20549 | *N. spathulata* | Soil,Tawain | EF669803 | EF669872 | - | [1] |
| CBS 483.65 | NRRL 5034 | *N. spinosa*, T | Soil, Nicaragua | EF669844 | EF669914 | EF669988 | [1] |
| CBS 498.65 |  | *N. stramenia,* T | Soil, USA | AY870766 | AY870726 | - | [5] |
| CBS 407.93 | IBT 21589 | *N. tatenoi,* T | Soil, Brazil | DQ114130 | DQ114139 | - | [7] |
| NRRL 35723 |  | *N. warcupi*, T | Soil, Australia | EU220283 | EU220284 | - | [9] |

* T, type strain

References:

1. Espinel-Ingroff A, Fothergill A, Fuller J, Johnson E, Pelaez T, et al. (2011) Wild-Type MIC Distributions and epidemiological cutoff values for caspofungin and *Aspergillus* spp. for the CLSI broth microdilution method (M38-A2 Document). Antimicrob agents chemother 55: 2855-2859.
2. Peterson SW (2008) Phylogenetic analysis of *Aspergillus* species using DNA sequences from four loci. Mycologia 100: 205-226.
3. Hong SB, Go SJ, Shin HD, Frisvad JC, Samson RA (2005) Polyphasic taxonomy of *Aspergillus fumigatus* and related species. Mycologia 97: 1316-1329.
4. Yaguchi T, Horie Y, Tanaka R, Matsuzawa T, Ito J, et al. (2007) Molecular phylogenetics of multiple genes on *Aspergillus* Section *Fumigati* isolated from clinical specimens in Japan. Jap J Med Mycol 48: 37-46.
5. Varga J, Due M, Frisvad JC, Samson RA (2007) Taxonomic revision of *Aspergillus* section *Clavati* based on molecular, morphological and physiological data. Stud Mycol 59: 89-106.
6. Balajee SA, Gribskov J, Hanley E, Nickle D, Marr K (2005) *Apsergillus lentulus* sp. nov., a new sibling species of *A. fumigatus*. Eukaryot Cell 4: 625-632.
7. Hong SB, Cho HS, Shin HD, Frisvad JC, Samson RA (2006) Novel *Neosartorya* species isolated from soil in Korea. Int J Syst Evol Microbiol 56: 477-486.
8. Hong SB, Shin HD, Hong J, Frisvad JC, Nielsen PV, et al. (2008) New taxa of *Neosartorya* and *Aspergillus* in *Aspergillus* section *Fumigati*. Antonie Van Leeuwenhoek 93: 87-98.
9. Samson RA, Hong S, Peterson SW, Frisvad JC, Varga J (2007) Polyphasic taxonomy of *Aspergillus* section *Fumigati* and its teleomorph *Neosartorya*. Studies in Mycology 59: 147-203.
10. Geiser DM, Frisvad JC, Taylor JW (1998) Evolutionary relationships in *Aspergillus* section *Fumigati* inferred from partial beta-tubulin and hydrophobin DNA sequences. Mycologia 90: 831-845.
